# ZamYOLO-maize: a YOLOv8n-based deep learning framework for automated detection and classification of maize leaf diseases in field conditions in Zambia

**DOI:** 10.3389/frai.2026.1764283

**Published:** 2026-02-25

**Authors:** Prudence Kalunga, Douglas Kunda, Aaron Zimba

**Affiliations:** 1 Computer Science Department, ZCAS University, Lusaka, Zambia; 2 DMI St Eugene University, Lusaka, Zambia; 3 Computer Science Department, ZCAS University, Lusaka, Zambia

**Keywords:** deep learning in agriculture, maize disease detection, object detection, precision agriculture, YOLOv8n

## Abstract

Maize, a critical staple crop in Zambia, faces persistent threats from foliar diseases such as Gray Leaf Spot, Northern Corn Leaf Blight, and Maize Streak Virus, significantly affecting smallholder productivity. Limited access to expert diagnostics, coupled with complex field conditions including occlusions and variable lighting, necessitates accessible, real-time disease detection systems tailored to local environments. To address this gap, this study first developed a novel field-captured dataset of Zambian maize leaf images, annotated with bounding boxes for disease lesions and labeled by disease type and severity to reflect real-world agri-ecological variability. Building on this dataset, we propose ZamYOLO-Maize, a multi-stage automated diagnostic framework integrating lesion detection, hierarchical disease classification, and severity assessment. A comparative evaluation was conducted using four state-of-the-art object detection models: YOLOv5n, YOLOv8s, YOLOv10s, and YOLOv8n, with performance assessed using precision, recall, F1-score, and inference speed. Experimental results demonstrate that YOLOv10s achieved the highest predictive performance (Precision = 0.997, Recall = 0.999, F1-score = 0.999), while YOLOv8n provided the optimal trade-off for edge deployment, achieving the fastest inference speed (4.65 ms/image) with a competitive F1-score of 0.995. The framework exhibited strong robustness under field variability, confirming its practical applicability. By integrating a locally representative dataset with an efficient deep learning pipeline, this study establishes a scalable foundation for mobile-based maize disease diagnostics, contributing to precision agriculture and supporting food security initiatives in Zambia and comparable agricultural regions.

## 1 Introduction

Maize (*Zea mays*) is the principal staple crop in Zambia, fundamental to national food security and the livelihoods of millions of smallholder farmers. The production of maize is highly affected by numerous viruses, viroids, fungi, and bacteria which are the primary causes of maize diseases. Discoloration, rot, scab, blight, necrosis, wilting, and abnormalities are common signs of infection and are used to identify foliar diseases in maize (Masood et al., 2023). Traditional disease detection in Zambia relies on manual scouting, a method that is inherently subjective, slow, and hampered by a scarcity of expert pathologists in rural areas (Kalunga and Kunda, 2025; Rua et al., 2023). This leads to a critical “detection latency,” where interventions are often applied too late to prevent significant yield loss (Mogili and Deepak, 2018). Masood et al. (2023) states that the accurate detection of maize leaf diseases is currently a crucial guarantee for maize yield for farmers without professional knowledge.

The manual leaf inspection method used in the traditional method of identifying maize diseases relies on the expertise of agricultural specialists and their understanding of plant pathology. Ineffective pesticide treatments, which not only contaminate the environment but also worsen the damaging effects on maize, are usually the consequence of misinterpreting the disease (Tian et al., 2019; Shen et al., 2025; Kamaleshkanna et al., 2024). Smallholder farming techniques, which are typified by inexpensive hardware, restricted access to professional plant pathology services, and extremely changeable field conditions, such as unregulated lighting, background clutter, and mixed symptom expression, dominate Zambia’s maize output. Therefore, to monitor the maize crops and cure the diseases, quick and precise methods are needed. There is a clear and urgent need for rapid, accurate, and scalable diagnostic tools to empower farmers and extension officers with timely information.

Plant disease monitoring and forecasting have recently made extensive use of digital technologies such as remote sensing, global positioning, and geographic information systems (Liu and Wang, 2021; Akhter and Sofi, 2022). Traditional plant disease diagnostic techniques are gradually being replaced by automated approaches based on computer vision and machine learning (ML) algorithms due to the significant advancements in artificial intelligence (Vishnoi et al., 2021). A few automated plant disease detection techniques based on digital images have been presented recently as a possible substitute for manual examination (Ngugi et al., 2021). Agriculture first used machine learning techniques with manually created features to enhance decision-making. Many current models are trained and validated on datasets collected under controlled or semi-controlled conditions, which restricts their applicability in actual African agricultural situations, despite recent advancements in deep learning-based plant disease identification.

Gray level co-occurrence matrix (GLCM) (Kaur et al., 2018), local binary patterns (LBP) (Pantazi et al., 2019), scale-invariant feature transform (SIFT) (Chouhan et al., 2021), histogram of oriented gradient (HOG) (Wani et al., 2021), and other methods are used in previous work as feature descriptors for the description of the images. These methods provide a simplified depiction of the plant disease by extracting visual features such as shape, hue, structure, and other statistical properties (Thakur et al., 2022). To classify leaf diseases, the collected characteristics are subsequently used to train machine learning models such decision trees (DT) (Panigrahi et al., 2020), support vector machines (SVM) (Chung et al., 2016; Aravind et al., 2018), and artificial neural networks (ANN) (Patil et al., 2017). Hand-coding feature calculation methods involve human knowledge and take a lot of processing time, even if they are simple to use and use little data. Deep learning (DL) has emerged as a transformative technology in precision agriculture, demonstrating exceptional capability in automating plant disease detection from leaf imagery (Barbedo, 2019a; Li et al., 2021).

Convolutional Neural Networks (CNNs) have repeatedly achieved high accuracy in controlled settings (Kamilaris and Prenafeta-Boldú, 2018). However, the translation of these technologies from research to practical field deployment in countries like Zambia faces significant hurdles. Recent developments in deep learning (DL) and artificial intelligence (AI) have made it possible to create automated plant disease detection frameworks that are faster and more objective than conventional methods. In controlled settings, leaf diseases have been accurately classified using convolutional neural networks (CNNs) and contemporary object-detection architectures. For instance, on gadgets like mobile phones and embedded computers, lightweight YOLO-based models have been used to identify leaf disease symptoms in real time (Ahmad et al., 2024; Tang et al., 2023; Ali et al., 2023; Wang et al., 2024).

Despite these developments, there are still large gaps in the application of these technologies to Zambia’s smallholder maize systems: Firstly, a lot of current research relies on publicly accessible or laboratory-grade datasets like Plant Village, which are unable to represent the noise and unpredictability present in actual field settings (Barbedo, 2019a; Li et al., 2021). When applied to real-world farming situations, models developed on such datasets frequently have poor generalizability. Secondly, there are few region-specific databases that accurately capture the agronomic and environmental realities of Zambia and other sub-Saharan African nations, where crop conditions and disease prevalence are different from those seen in global benchmarks (Kamilaris and Prenafeta-Boldú, 2018). Third, even though high-performing deep learning architectures have been developed, many of them are computationally demanding and inappropriate for implementation in settings with limited resources, where farmers and extension agents do not have access to sophisticated hardware (Kalunga and Kunda, 2025; Ferentinos, 2018). The “black box” nature of complex models hinders adoption, as end-users require transparent and interpretable predictions to trust and act upon the AI’s recommendations (Wu et al., 2020; Adadi and Berrada, 2018).

The environmental complexity found on actual farms such as uneven lighting, occlusion, and mixed infections is absent from laboratory or benchmark datasets used in the development and validation of many current models (Chen et al., 2023; Singh et al., 2022). Instead of concentrating on maize in African field circumstances, most of the lightweight detection or DL (deep learning) models concentrate on detecting generic leaf diseases or other crops. To enable deployment on resource-constrained platforms utilized by farmers and extension agents, it is imperative to strike a compromise between detection accuracy and computing efficiency. Although better YOLO variations (like YOLO-DBW) have been developed, they frequently raise hardware requirements or complexity. To support scalable, farmer-centered diagnostic tools and enable more accurate disease detection under natural field conditions, a locally curated dataset and validation framework reflecting the realities of Zambian smallholder farms was developed. Thus, the purpose of this study was to create and validate ZamYOLO-Maize, a lightweight deep learning framework for the in-field, real-time detection of maize leaf diseases. The following research questions were specifically addressed by the study:

1. Does the suggested YOLOv8n-based framework detect and categorize key maize leaf diseases with state-of-the-art accuracy in a variety of Zambian field conditions?
2. How does the model compare to existing deep learning algorithms in terms of precision, recall, and on-device inference speed for real-time deployment?
3. Is the framework resilient to common field challenges such as complex backgrounds, partial occlusions, and variable lighting?

The paper proposes ZamYOLO-Maize, a lightweight, field-adapted deep learning system built on theYOLOv8n object detection architecture (You Only Look Once version 8 nano), as a solution to these problems. For resource-constrained agricultural situations where deployment on mobile devices or edge-computing platforms is crucial, the YOLOv8n model was chosen because of its ideal balance of accuracy and computational economy (Khan et al., 2023). Its nano offers cutting-edge performance with low processing requirements and real-time inference, which are essential features for field deployment. This paper proposes a deep learning-based framework for maize disease detection that is specifically designed for the challenges of the Zambian smallholder context. This work’s distinctiveness resides in a few distinctive scientific and practical advances that go far beyond optimizing an already-existing YOLO architecture: The key contributions of our work are:

1. We present a new field-captured dataset of photos of maize leaves that are uniquely labeled with severity labels, disease type classifications, and bounding boxes for disease lesions. This dataset addresses the significant shortage of region-specific, multi-tier agricultural data and serves as a benchmark for future research in automated plant disease identification.
2. We propose ZamYOLO-Maize, a comprehensive deep learning framework that includes optimal lesion identification, hierarchical disease classification, severity estimate, and a rule-based treatment advising system. This is the first comprehensive automated diagnostic pipeline designed specifically for maize diseases in smallholder farming settings.
3. Through extensive comparative and ablation studies, we demonstrate that our YOLOv8n-based detector achieves an optimal balance of high accuracy (F1-score > 0.995) and real-time inference speed (4.65 ms/image), proving its practical viability for resource-constrained, mobile-based deployment in field settings.

ZamYOLO-Maize seeks to offer a workable solution for real-time, scalable disease diagnosis in smallholder maize farming systems in Zambia by combining field-relevant data with a computationally effective detection model, thereby improving crop management and food security. The rest of the paper is arranged as follows: Section II examines previous studies related to identifying diseases in plants, specifically maize crop diseases. In Section III, the adopted methodology and detailed architecture of the proposed framework are explained, details of the selected dataset, implementation, and experimental setup. Section IV presents the results obtained and discussion. Lastly, we concluded our work and suggested some future directions in Section V.

## 2 Related works

In this section, we review the literature on deep learning-based maize disease detection and classification, with an emphasis on YOLO-based techniques and other lightweight strategies. We outline the main developments, their drawbacks, and how ZamYOLO-Maize either builds on them or differs from them.

Deep learning has been used in several research to identify maize diseases using CNN-based categorization. Using both deep (ResNet) and lightweight (MobileNet) architectures, (Barbedo, 2019a) created a two-stage transfer learning technique. Their research showed that even when training data originates from controlled datasets like PlantVillage, transfer learning on field-collected maize leaf photos may achieve excellent accuracy (up to 99.11% on MobileNet). Mogili and Deepak (2018) developed a Dense CNN model that effectively classified maize leaf diseases, achieving over 98% accuracy on controlled datasets. However, both approaches relied on non-field datasets, limiting their adaptability to real-world farming conditions. Li et al. (2021) explored hyperspectral imagery for detecting maize leaf spot, demonstrating the potential of spectral features to capture subtle disease symptoms. Although hyperspectral methods offer high precision, their cost and complexity restrict use in smallholder farming systems. Kamilaris and Prenafeta-Boldú (2018) introduced a ShuffleNetV2-based CNN optimized for lightweight mobile deployment, highlighting a growing shift toward efficiency-focused architectures for field applications. Jiang et al. (2024) used MobileNetV2 to create a CNN-based system for classifying maize diseases. Although their approach focuses on deployment in resource-constrained situations and targets many leaf disease classes (such as Common Rust, Gray Leaf Spot, and Blight), it manages whole-image classification instead of explicit object localization.

Despite their success in recognition, these classification-based methods frequently lack the ability to precisely localize (bounding boxes), which is crucial for comprehending the distribution of diseases on leaves and for focused therapies (Mohanty et al., 2016).

By automatically learning hierarchical feature representations from raw pixel data, deep learning in particular, Convolutional Neural Networks (CNNs) overcame many of the drawbacks of conventional techniques, signaling a paradigm shift in the area. Using the PlantVillage dataset, the seminal study by Li et al. (2021) showed that CNNs such as AlexNet and GoogLeNet could classify 26 crop-disease pairs with expert-level accuracy, much exceeding conventional approaches (Mogili and Deepak, 2018).

Further studies solidified CNNs’ hegemony. Transfer learning, which involves fine-tuning models pre-trained on large-scale datasets like ImageNet on smaller plant disease datasets, has becoming commonplace. Ferentinos (2018) investigated a few deep CNN architectures and discovered that on the PlantVillage dataset, VGG and ResNet models could attain over 99% accuracy (Barbedo, 2019a). According to Barbedo (2019b), who examined the extensive success of CNNs in several agricultural areas (Li et al., 2021), the use of these models went beyond classification to encompass segmentation and severity estimation. However, models trained on laboratory-grade photos (e.g., PlantVillage) frequently fail to generalize to field circumstances, revealing a substantial “domain shift” problem (Kamilaris and Prenafeta-Boldú, 2018). This is a perennial criticism, as mentioned by (Liu et al., 2018).

Object detection offers a more detailed analysis by both categorizing and localizing several disease cases inside a single image, whereas classification models give a single label to an entire image. In real-world situations, where leaves may display several diseases or symptoms at various times, this is essential. When these models are evaluated using field-acquired imagery, empirical evaluations have demonstrated significant declines in classification and detection accuracy, especially in smallholder scenarios where symptoms vary in severity and co-occur with environmental noise (Barbedo, 2019a; Arsenovic et al., 2019). These results emphasize the necessity of geographically and agronomically representative datasets to enhance model generalization and assist practical agricultural decision-making, particularly for underrepresented regions like Sub-Saharan Africa.

Two-stage detectors, like Faster R-CNN, which first produce region recommendations and then categorize them, were used in agriculture’s early acceptance. For instance, Barbedo (2019b) employed a Faster R-CNN to accurately identify apple diseases. However, these models’ computational complexity frequently led to sluggish inference times.

A better speed-accuracy trade-off was provided by the following move to one-stage detectors, which carry out localization and classification in a single pass. Adoption of models such as the You Only Look Once (YOLO) family and Single Shot MultiBox Detector (SSD) was rapid. Early iterations, such as YOLOv3, demonstrated real-time capabilities that two-stage detectors lacked and were effectively used to identify pests and diseases in crops like rice and tomatoes (Pan et al., 2025; Wang et al., 2021; Liu and Wang, 2020). This tendency was supported by a thorough analysis of object detection advancements by (Arsenovic et al., 2019; Kamaleshkanna et al., 2024), which noted the growing inclination for one-stage detectors in real-time performance-demanding applications.

Object detection frameworks like YOLO (You Only Look Once), SSD (Single Shot Detector), and Faster R-CNN have gained popularity due to their capacity to concurrently localize and categorize several diseases, whereas most of the previous research concentrated on image-level classification (Ferentinos, 2018; Shen et al., 2025). Khan et al. (2023) used YOLO-based models to identify maize leaf disease, and on a mobile-based system, they recorded an accuracy of 99.04% using YOLOv8n. Nevertheless, their dataset lacked the environmental diversity typical of smallholder farms in Africa and was not region-specific. An enhanced YOLOv8 model was suggested by Yang et al. (2024) to identify maize leaf spot disease in actual field settings. To improve feature extraction, their version integrates a Global Attention Mechanism (GAM) and a Slim-neck module into the network. In real-world circumstances, their upgraded version outperformed baseline YOLOv8 with precision of 95.18%, recall of 89.11%, and mAP@50 of 94.65. Similarly, Waheed et al. (2020) presented YOLOv8-GO, a lightweight version of YOLOv8 that has an omni-dimensional dynamic convolution (ODConv) module and an additional Global Attention Mechanism before the SPPF layer. With a mAP@50 of 88.4% and a very high FPS, this architecture strikes a balance between accuracy and computational cost, making it suitable for real-time field detection. Recent object-detection research has progressed rapidly: Ultralytics’ YOLOv8 has become widely adopted for real-time applications due to its efficient backbone and streamlined training utilities, and newer YOLO family variants (e.g., YOLOv9) propose architectural and training improvements such as programmable gradient information (PGI) and GELAN for improved parameter utilization (Jocher et al., 2023; Singh et al., 2022).

Concurrently, transformer-based detectors (DETR and its variations) have shown great performance in complex settings, although they frequently incur higher computing cost and longer training times, making YOLO variants still suitable for mobile agricultural deployments (Carion et al., 2020). Several recent studies have already deployed YOLOv8 to plant disease diagnosis, revealing promising mAP and inference-time trade-offs under field settings, which highlights the significance of our Zambia-specific evaluation and optimization efforts (Chen et al., 2023; Ali et al., 2023).

These YOLO-based methods demonstrate how the trade-off between accuracy and inference speed can be improved by architectural improvements (attention, efficient convolution, feature fusion). But most of the current work is constrained in the following ways: They frequently optimize for performance rather than balancing computational efficiency for edge deployment; (1) they might not have been validated on datasets that accurately reflect the variability of smallholder farms in sub-Saharan Africa; and (2) they might not have undergone rigorous field testing. Other studies that focus on lightweight deep learning for plant disease detection go beyond YOLO.

For instance, Osouli et al. (2022) presented a successful method for identifying maize diseases by integrating pre-trained MobileNetV2 and Inception networks with data augmentation and transfer learning. On a small amount of training data, they reported an accuracy of about 97%.

Explainable models that integrate CNN topologies with Vision Transformers have been developed, albeit they are not exclusive to maize. For example, the PlantXViT model employs a hybrid CNN-ViT architecture to identify plant diseases (including maize) while offering interpretability using methods like Grad-CAM and LIME. In agricultural settings, this explainability is beneficial for acceptance and confidence, but it usually comes with a higher computational cost. Although two-stage architectures like Faster R-CNN, which frequently achieve excellent accuracy, have been used in previous research, their computational expense makes them unsuitable for the real-time, in-field deployment scenarios that are the focus of contemporary precision agriculture (Tang et al., 2023).

The accuracy gap has been reduced while preserving remarkable speed because of the recent development of one-stage detectors, especially the YOLO family (Wu et al., 2020). From version 5 to the most recent version 10, the YOLO architecture has experienced fast iteration, with advancements in neck architecture, backbone design, and training techniques that improve efficiency and performance (Adadi and Berrada, 2018; Arrieta et al., 2020). For the Zambian context and comparable settings, where solutions must operate on inexpensive, resource-constrained hardware, this emphasis on efficiency is essential (Redmon et al., 2016). To meet the urgent need for solutions that are both accurate and practically deployable, this work purposefully concentrates on benchmarking the most recent and effective models from the YOLO family, namely YOLOv5, v8n, v8s, and v10. This paper offers crucial insights for choosing the best architecture for edge deployment in actual agricultural settings by performing a thorough comparison analysis of these models using a maize disease dataset unique to Zambia.

Three primary research gaps are apparent from the evaluated literature: 1. Even when they are accurate, a lot of models are trained on datasets that do not accurately represent African smallholder farming settings. 2. Even though YOLOv8 and other detection models are strong, implementation on mobile or edge devices requires additional optimization (attention modules, lightweight convolutions, effective featurefusion).

Spatial localization, which is essential for mapping disease severity and directing therapies, is absent from pure classification models (CNN-based). 3. Few maize disease models presently meet the need for models that can explain their predictions to agronomists and farmers, which is necessary for the adoption of AI in agriculture.

ZamYOLO-Maize sets itself apart by specifically filling in these gaps: it uses YOLOv8n for computational efficiency, integrates design decisions to maintain high accuracy while being deployable on edge/mobile devices, and is refined on a field-collected maize leaf dataset unique to Zambia. Through a balanced, useful detection system, it seeks to promote trust and acceptance by local farmers and extension agents, in contrast to most previous works.

## 3 Methodology

This section covers the methodological framework utilized in building and assessing ZamYOLO-Maize, an upgraded deep-learning model for automated maize leaf disease detection under Zambian field settings. Strict dataset preparation, a repeatable training process, architectural advances, statistical analysis, ablation investigations, and comparative benchmarking against cutting-edge detectors are all incorporated into the methodology.

### 3.1 Data set preparation

The collection includes annotated images of four different maize leaf conditions: Northern Leaf Blight (NLB), Gray Leaf Spot (GLS), Maize Streak Virus (MSV), and Healthy. Images were taken under varied real-field situations in Zambia including changeable illumination, shadows, occlusions, and complicated backgrounds to improve model robustness in practical deployments. All images were manually inspected for quality, and corrupted or ambiguous samples were eliminated. Bounding boxes were annotated in YOLO format (𝑥𝑐𝑒𝑛𝑡𝑒𝑟, 𝑦𝑐𝑒𝑛𝑡𝑒𝑟, 𝑤𝑖𝑑𝑡h, ℎ𝑒𝑖𝑔ℎ𝑡) normalized to [0,1].

**Figure 1:**
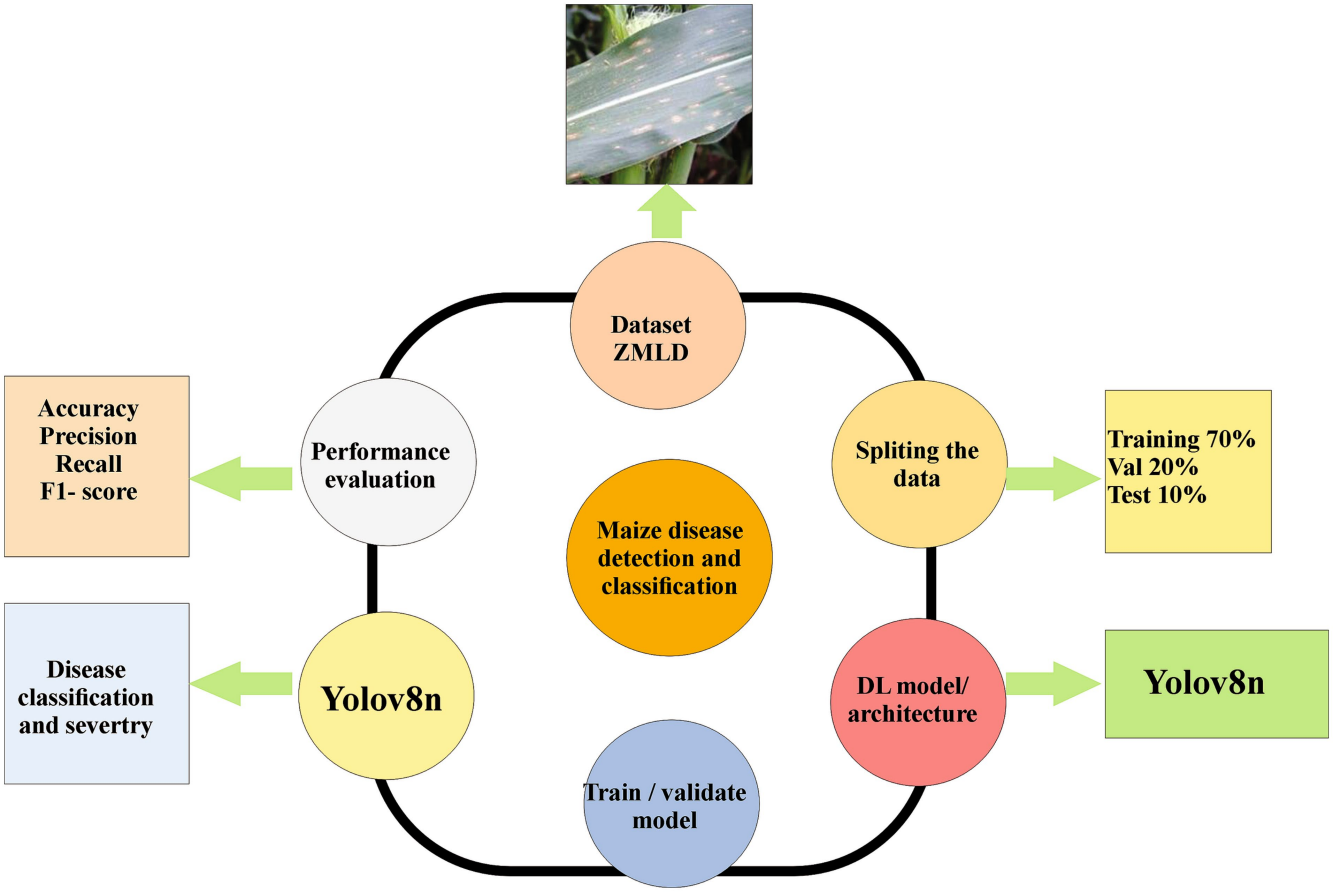
System workflow from raw in-field image acquisition → annotation & preprocessing → YOLOv8n training → model inference & deployment.

To ensure a robust and unbiased evaluation, the dataset was partitioned into:

70% Training set which was used for model learning.
20% Validation set which was used for hyperparameter tuning and early stopping.
10% Test set was held out set for final performance evaluation (Figure 1).

To maintain distributional balance across disease categories, splits were carried out using class-stratified sampling. Images of maize leaves from farms in Zambia’s Lusaka, Central, Eastern, and Northern provinces were gathered to create a bespoke dataset. There are three main disease classes in the dataset. Mobile phone cameras were used to take pictures in the field to replicate actual operational settings with fluctuating lighting, foliage occlusions, motion blur caused by wind, and mixed disease signs. The final dataset consists of 19,990 images spread throughout the four classes. Gray Leaf Spot (GLS) 5,000 samples, Northern Leaf Blight (NLB) 4,990 samples, and Maize Streak Virus (MSV) 5,000, Healthy 5,000 samples. The three being the main disease classes that frequently impair Zambian maize output. Images were taken at various times of day, plant growth stages, and farm management techniques to guarantee diversity.

The lack of localized maize disease datasets in sub-Saharan Africa, where environmental circumstances are significantly different from controlled datasets like PlantVillage, is addressed by this region-specific dataset. The Zambia Maize Leaf Dataset, a publicly accessible annotated dataset with 19,990 photos in four classes, was used for the experiments (Kalunga, 2026; Table 1).

**Table 1:** Summary of image collection details.

Category	Details
Camera models	Canon EOS Rebel T7 DSLR Camera with 18-55 mm Lens. Samsung A10 Phone: 6.2-inch HD + Infinity-V Display with a 720 × 1,520 resolution; 155.6 mm × 75.6 mm × 7.9 mm and weighs 168 g; 32 GB internal storage, expandable up to 512 GB via MicroSD and 2 GB RAM. It has a non-removable 3,400 mAh battery
Temporal distribution	Images captured at various times of day; precise timestamp embedded in image metadata.
Growth stages	Multiple crop growth stages represented varying between 1to 4 months
Geographic distribution	Lusaka, Central, Northern, and Eastern provinces.

The collection includes photos taken in four provinces (Lusaka, Central, Northern, and Eastern) by agriculture extension officers using Samsung smartphones and Canon cameras. The photos, which have metadata timestamps attached to them, show different times of the day when they were captured. The different stages of crop growth, range from one to 4 months.

### 3.2 Dataset annotation

A Python script was created to annotate the dataset and create bounding boxes around disease areas for each image using the local machine using Cursor IDE. Algorithm 1 was used to collect the raw YOLO-format annotations and transform them into a structured YAML database. Each YOLO annotation file is methodically processed by this program, which also arranges all bounding box coordinates into a single format and translates numerical class IDs to human-readable disease names therefore creating an annotations.yaml file. The yaml file facilitates effective dataset loading and preprocessing in later model training by offering a machine-readable index connecting each image to its full set of disease lesion annotations.

The performed steps for dataset annotation aggregation and formatting are shown in Algorithm 1 below:

ALGORITHM 1 Dataset annotation aggregation and formatting.Initialize empty list dataset ← []
Define class_labels ← [‘gray leaf spot’, ‘maize streak virus’, ‘northern leaf blight’, ‘healthy’]
FOR each filename in LIST_FILES(yolo_labels_dir) DO
IF filename ends with ‘.txt’ THEN
image_name ← REPLACE(filename, ‘.txt’, ‘.jpg’)
image_path ← JOIN(images_dir, image_name)
annotation_list ← []
Open label_file ← OPEN(JOIN(yolo_labels_dir, filename))
FOR each line in READ_LINES(label_file) DO
Parse: class_id, x_center, y_center, width, height ←
SPLIT(line) and CONVERT_TO_FLOAT
Create annotation_entry ← {
‘class’: class_labels[INTEGER(class_id)],
‘x_center’: x_center,
‘y_center’: y_center,
‘width’: width,
‘height’: height}
APPEND annotation_entry to annotation_list
END FOR
CLOSE(label_file)
Create image_record ← {
‘image’: image_path,
‘annotations’: annotation_list}
APPEND image_record to dataset
END IF
END FOR
Open yaml_file ← OPEN(‘annotations.yaml’, write_mode)
YAML_DUMP(dataset, yaml_file, sort_keys = False)
CLOSE(yaml_file)
RETURN ‘annotations.yaml created successfully’

### 3.3 Data augmentation

Several augmentation strategies, including rotation, brightness/contrast modifications, random scaling, and horizontal and vertical flipping, were used to improve model generalization and boost dataset diversity. These additions improve resilience and mimic field variability.

### 3.4 Model architecture

**Figure 2:**
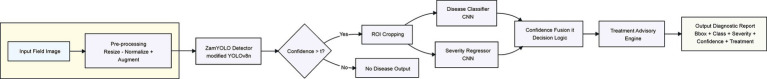
Detailed workflow of the ZamYOLO-Maize diagnostic framework showing decision logic and parallel processing.

The proposed ZamYOLO-Maize architecture is an end-to-end diagnostic pipeline designed to interpret field-captured maize leaf photos and provide full disease reports. As demonstrated in Figure 2 and detailed in Algorithm 2, the system includes sequential and parallel modules for detection, classification, severity evaluation, and treatment advice.

ALGORITHM 2 Decision logic and sequential steps of ZamYolo Maize workflow**Input**
: Raw maize leaf images
**Output:** Trained ZamYOLO-Maize model for deployment
        **Data Acquisition**        1.1 Collect maize leaf images from field conditions
        1.2 Store images in structured dataset folders
        **Data Preprocessing**        2.1 For each image in dataset:
          a. Resize image to 640 × 640 resolution
          b. Apply augmentation (flip, rotate, scale, color-jitter)
          c. Annotate bounding boxes and class labels
        2.2 Save processed images into Zambia Maize Leaf Dataset (ZMLD)
        **Model Initialization (YOLOv8n Base)**        3.1 Load YOLOv8n backbone
        3.2 Initialize model components:
          • Stem layer
          • Backbone
          • Neck (FPN + PAN)
          • Detection head
        **Model Training**        4.1 Split dataset into train/validation/test sets
        4.2 For each training epoch *E*:          a. Feed batch of preprocessed images
          b. Extract multi-scale features through backbone
          c. Fuse features through FPN/PAN
          d. Predict bounding boxes & class scores
          e. Compute losses (cls + box + obj)
          f. Backpropagate gradients
          g. Update model weights
        4.3 Evaluate performance using precision, recall, F1, and mAP@50
        **Model Optimization (Optional Enhancements)**        5.1 *If applying channel pruning:*          a. Identify low-importance channels
          b. Remove redundant channels
        5.2 *If applying knowledge distillation:*          a. Train student model using teacher predictions
        **Deployment**        6.1 Export trained ZamYOLO-Maize model
6.2 Deploy on mobile device for field-level inference
**End Algorithm**

**Figure 3:**
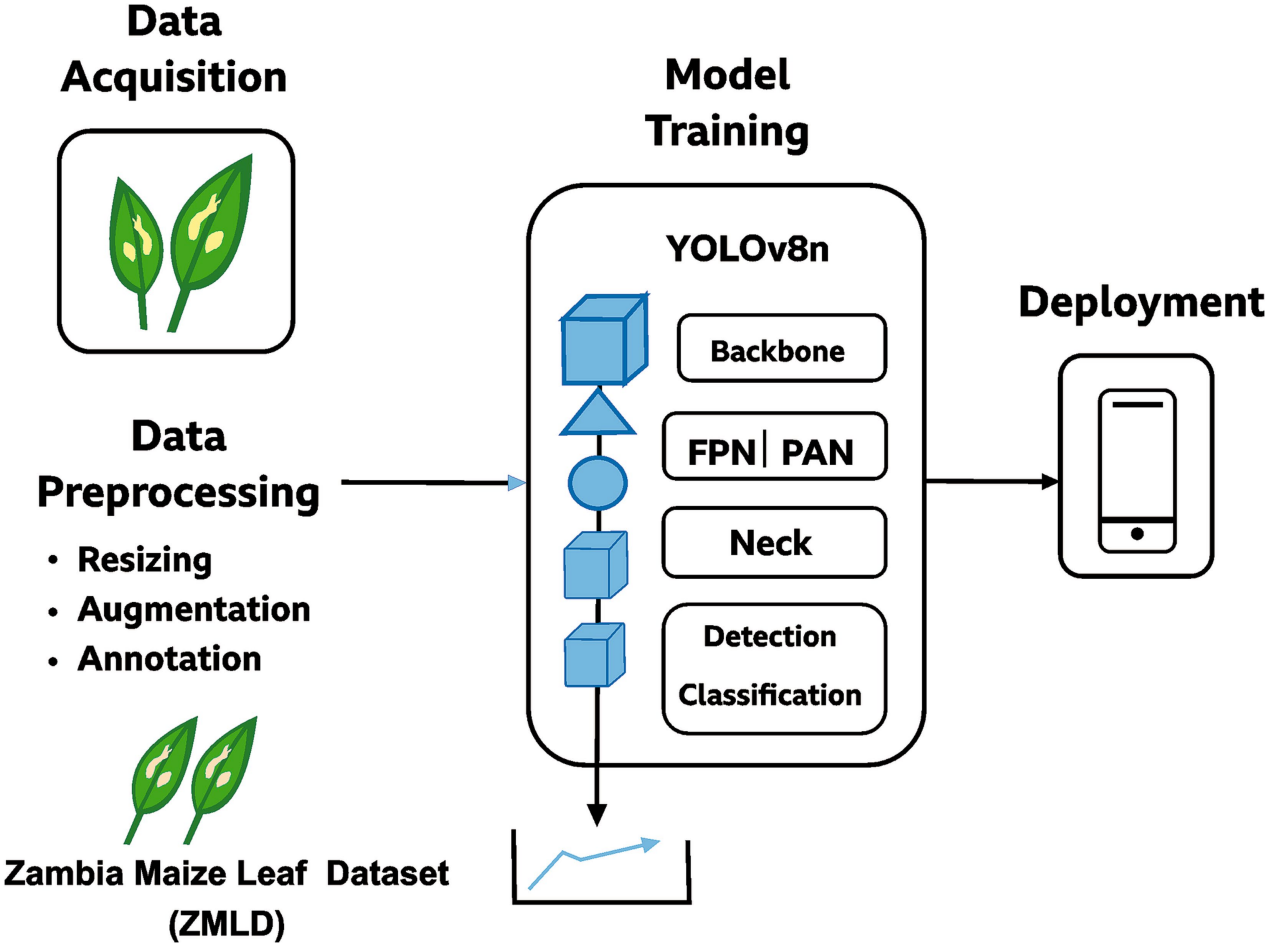
Schematic diagram of the proposed YOLOv8n-based maize leaf disease detection workflow.

**Figure 4:**
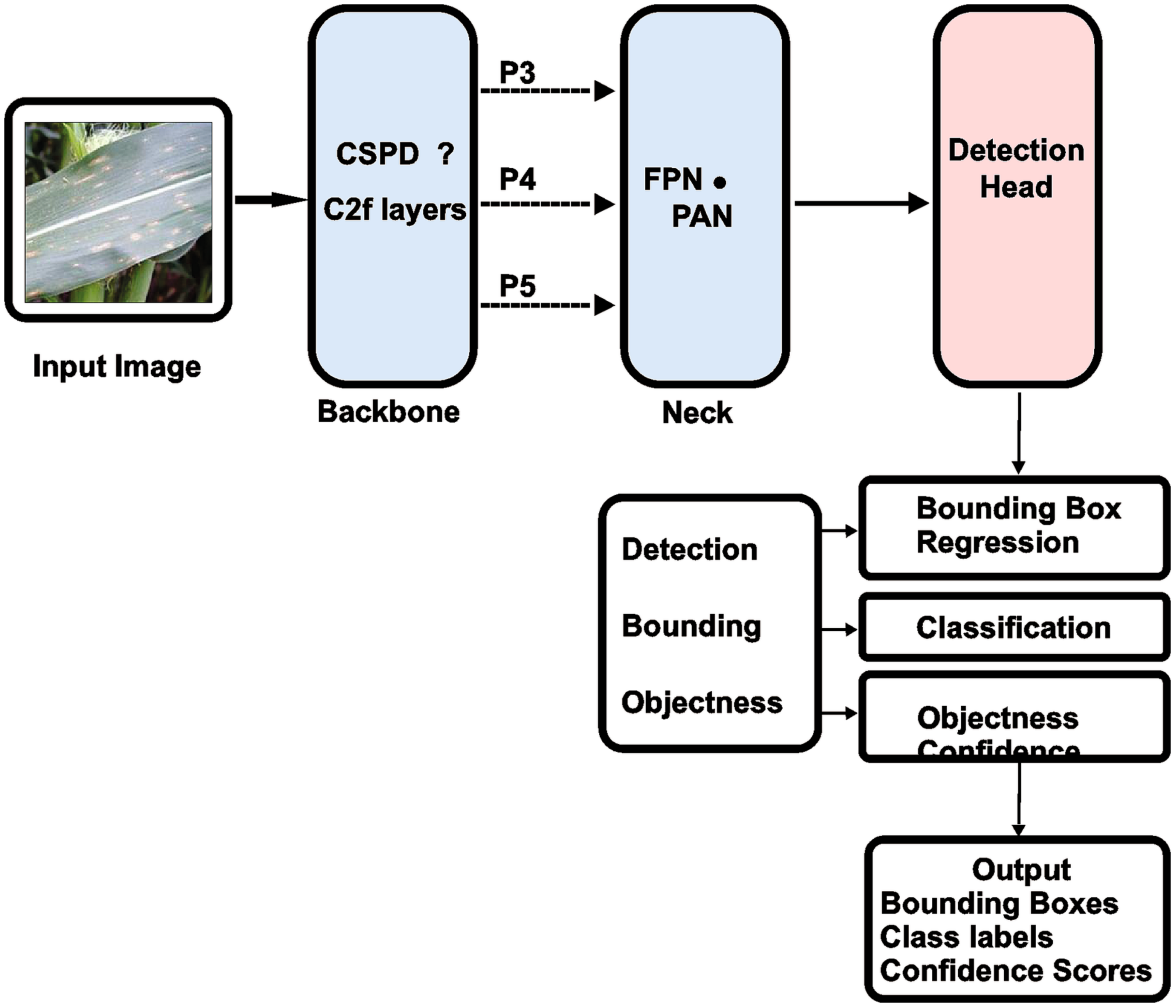
Architecture of the proposed YOLOv8n maize leaf disease detection model.

The best balance between detection accuracy and computational performance led to the selection of Ultralytics’ YOLOv8n architecture (Wang et al., 2021). The YOLOv8n-based architecture (ZamYOLO-Maize) consists of: Backbone (Feature Extraction), C2f modules, CSPDarknet structure, SPPF (Spatial Pyramid Pooling – Fast). The backbone extracts multi-scale features from raw images. The Neck (Feature Fusion), PANet + FPN, top-down and bottom-up feature fusion, produces P3, P4, P5 multi-scale output maps. The Detection Head, a decoupled detection head predicts, bounding box coordinates, objectness scores, class probabilities. This separation improves convergence speed and detection accuracy (Adadi and Berrada, 2018). YOLOv8n is perfect for small-farm deployments since it uses less GPU RAM and provides faster inference than heavier variants (YOLOv8m, YOLOv8x) (Figures 3, 4).

### 3.5 Loss functions

The total loss L_total used in YOLOv8n combines: CIoU Loss (Bounding Box Regression) Complete Intersection over Union (CIoU) improves bounding box accuracy by accounting for:

- IoU overlap
- Distance between center points
- Aspect ratio consistency

It accelerates convergence and improves localization in complex leaf shapes.

BCE Loss (Classification and Objectness).

Binary Cross-Entropy (BCE) is used for:

- Objectness prediction
- Class label prediction

BCE is preferred for multi-label environments and improves stability when detecting multiple diseases within a single leaf.

**Figure 5:**
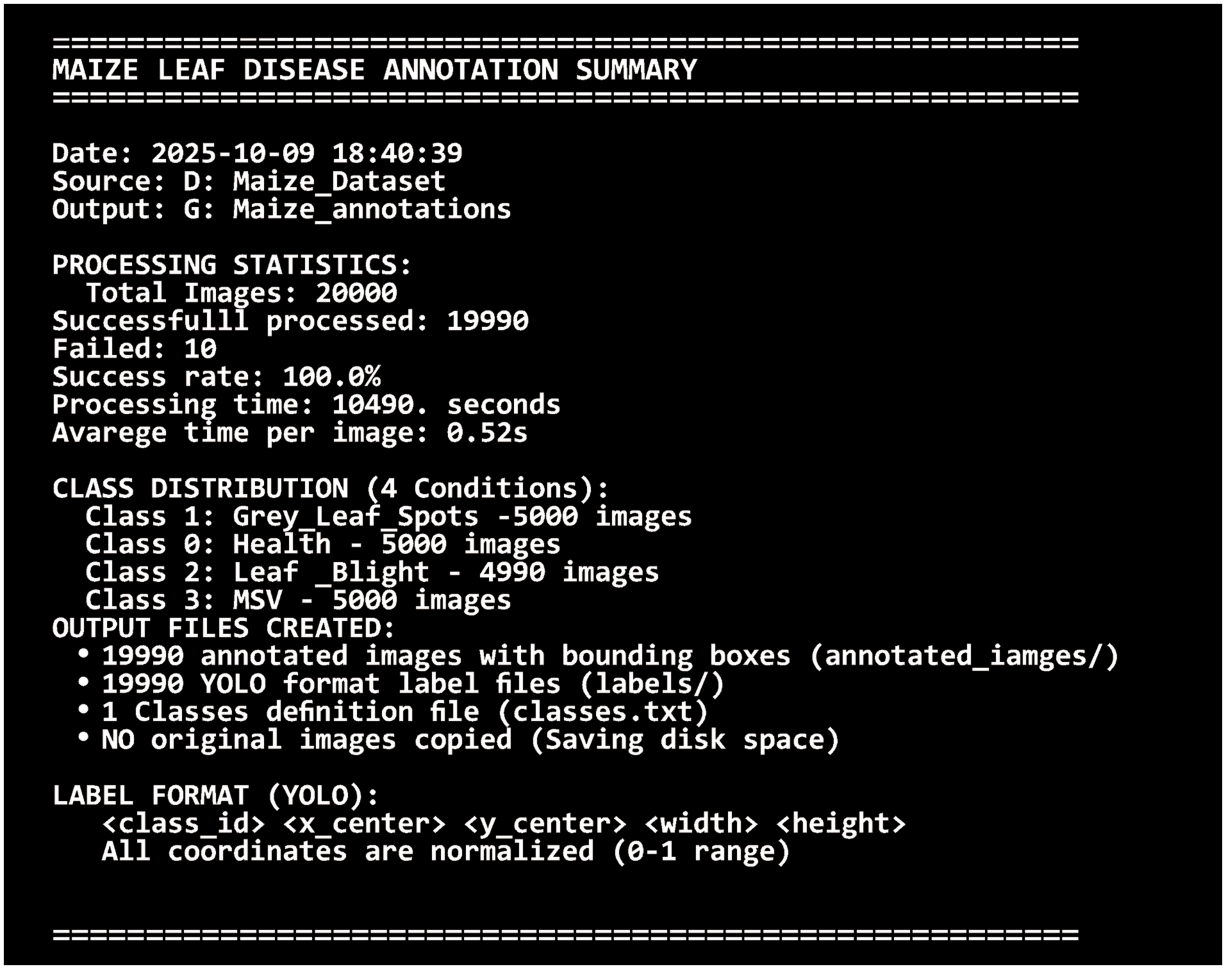
Detailed workflow of the ZamYOLO-Maize diagnostic framework showing decision logic and parallel processing. It should read as maize leaf notation summary.

Figure 5 demonstrates the architecture of the ZamYOLO-Maize framework. The pipeline starts with pre-processing field images, then uses our modified YOLOv8n model (ZamYOLO Detector) to detect lesions. A hierarchical CNN for disease classification and a regression network for severity assessment receive the detected areas of interest (ROIs) in simultaneously. The confidence scores from detection and classification are merged in a weighted decision module. Based on the combined disease kind and severity level, a rule-based therapy advising engine proposes specific treatment. The system provides a structured diagnostic report with bounding box coordinates, disease descriptions, severity scores, confidence metrics, and therapy recommendations.

### 3.6 Proposed framework and experimental setup

The YOLOv8n architecture, which is optimized for lightweight, real-time performance in resource-constrained agricultural applications, is the foundation of the suggested detection system, ZamYOLO-Maize.

#### 3.6.1 Training and optimization

We used the Yolov8n algorithm on the Zambian Maize Leaf Dataset, and the network was trained using, batch size, learning rate, and the number of epochs with the training optimizer to achieve the best results. The recommended ZamYolo-Maize framework was implemented using Python in Google Colab Pro, and all necessary packages and libraries were installed. Training was conducted using the Ultralytics YOLOv8 framework on a Google Colab Pro+ environment with NVIDIA A100 GPU. The annotated dataset was then exported in YOLO v8 format (YAML configuration with class names and image locations) after being split into subsets for training (70%), validation (20%), and testing (10%).

#### 3.6.2 Software and libraries

Python 3.10.12 (Google Colab default) was used to set up the experimental environment. Key libraries included OpenCV2 (OpenCV-python) for image processing, PyTorch (version 2.1.0 + cu118) as the core deep learning framework with support for CUDA 11.8, and Ultralytics YOLO (version 8.0.196) for model architecture and training. Seaborn, NumPy, Matplotlib, and pandas were tools for data processing and visualization. To manage the dataset, Roboflow and Kaggle packages were installed.

#### 3.6.3 Training hyper parameters

Early stopping and checkpointing (based on best validation mAP) were used to prevent over-fitting.

### 3.7 Evaluation metrics

The model’s performance was evaluated using standard object-detection metrics, with values of metrics such as precision score, recall score, F1-score, IoU score, and mean average precision (mAP) score, mAP@50–95 for each. Precision (P) is the ability to avoid false positives, Recall (R) is the ability to detect all true positives. F1-Score is the balance between P and R; mAP@50 is the mean Average Precision at 0.5 IoU. @50 refers to an IoU threshold of 0.50 (i.e., a predicted bounding box is considered correct if its Intersection over Union with the ground truth is at least 50%). mAP@50–95 is the mean Average Precision averaged over Intersection over Union (IoU) thresholds from 0.5 to 0.95 in steps of 0.05. IoU (Intersection over Union) measures the overlap between predicted and ground truth bounding boxes. It ranges from 0 to 1 (Table 2).


$$\mathrm{IoU}=\frac{\text{Area of overlap}}{\text{Area of Union}}$$

**Table 2:** Software libraries and versions used in the experimental environment.

Software/Library	Version	Purpose
Python	3.10.12	Primary programming language
PyTorch	2.1.0 + cu118	Core deep learning framework
Ultralytics YOLO	8.0.196	Object detection model training
OpenCV	(Latest from pip)	Image processing
CUDA Toolkit	11.8	GPU acceleration support
Pandas/NumPy	(Latest from pip)	Data manipulation and numerical operations
Matplotlib/Seaborn	(Latest from pip)	Results visualization

These metrics align with standard practices in object detection research and were computed as follows:


$$\text{Precision}=\frac{\mathrm{TP}}{\mathrm{TP}+\mathrm{FP}}$$

$$\text{Recall}=\frac{\mathrm{TP}}{\mathrm{TP}+\mathrm{FN}}$$

$$\text{Accuracy}=\frac{\mathrm{TP}+\mathrm{TN}}{\mathrm{TP}+\mathrm{TN}+\mathrm{FP}+\mathrm{FN}}$$

$$F1\;\text{Score}=\frac{P*R\times 2}{P+R}$$

**Figure 6:**
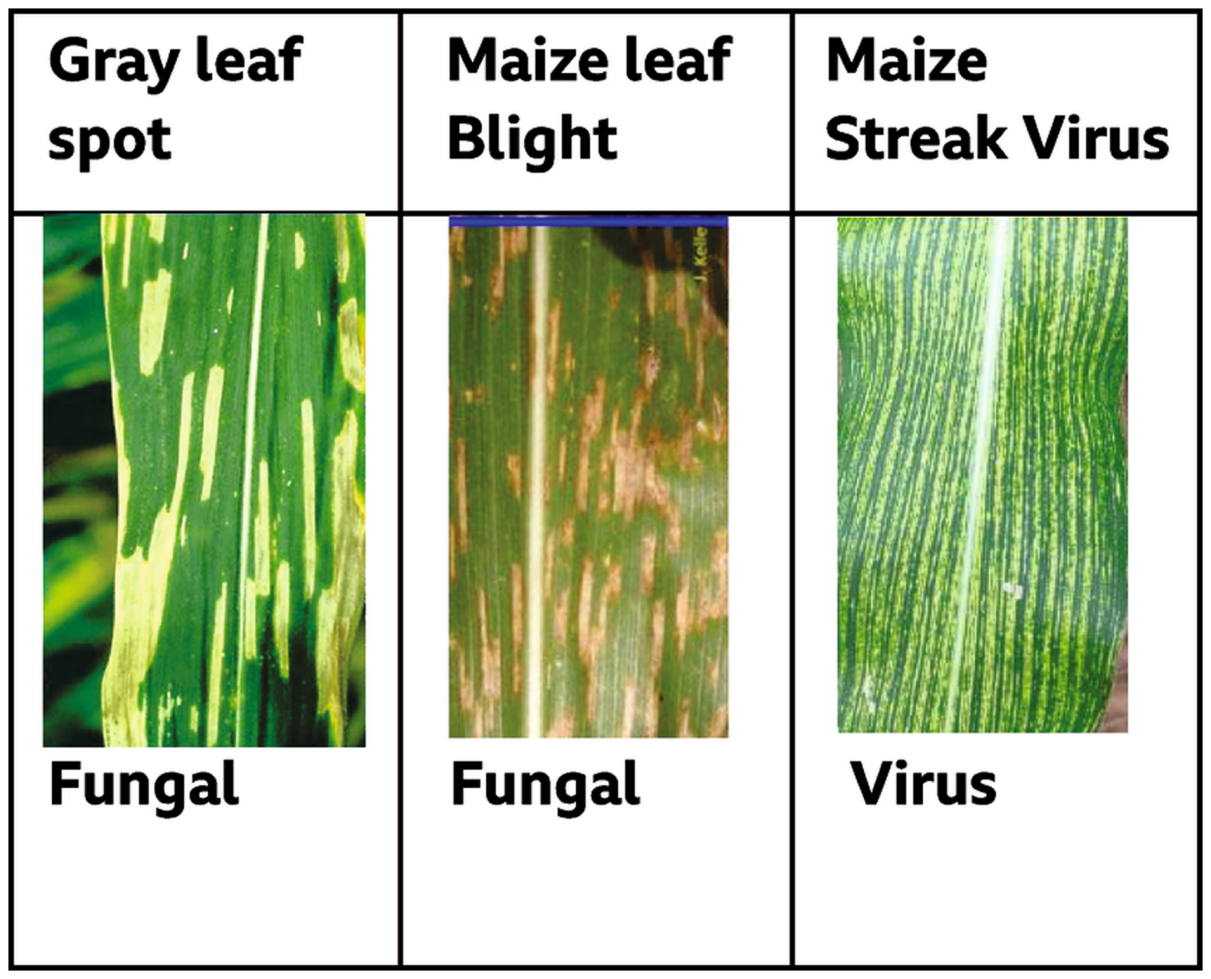
Samples from the three maize diseases.

In this case, TP stands for true positive score, which represents the total number of positive samples with the target disease class accurately categorized. The total number of negative samples with positive predictions is represented by the false positive score, or FP. The number of positive samples with incorrectly assessed disease classes is shown by the FN, which stands for false negative. Finally, the TN stands for true negative, which denotes the samples for which the model accurately predicted as the negative class (Figure 6).

### 3.8 Visual results

**Figure 7:**
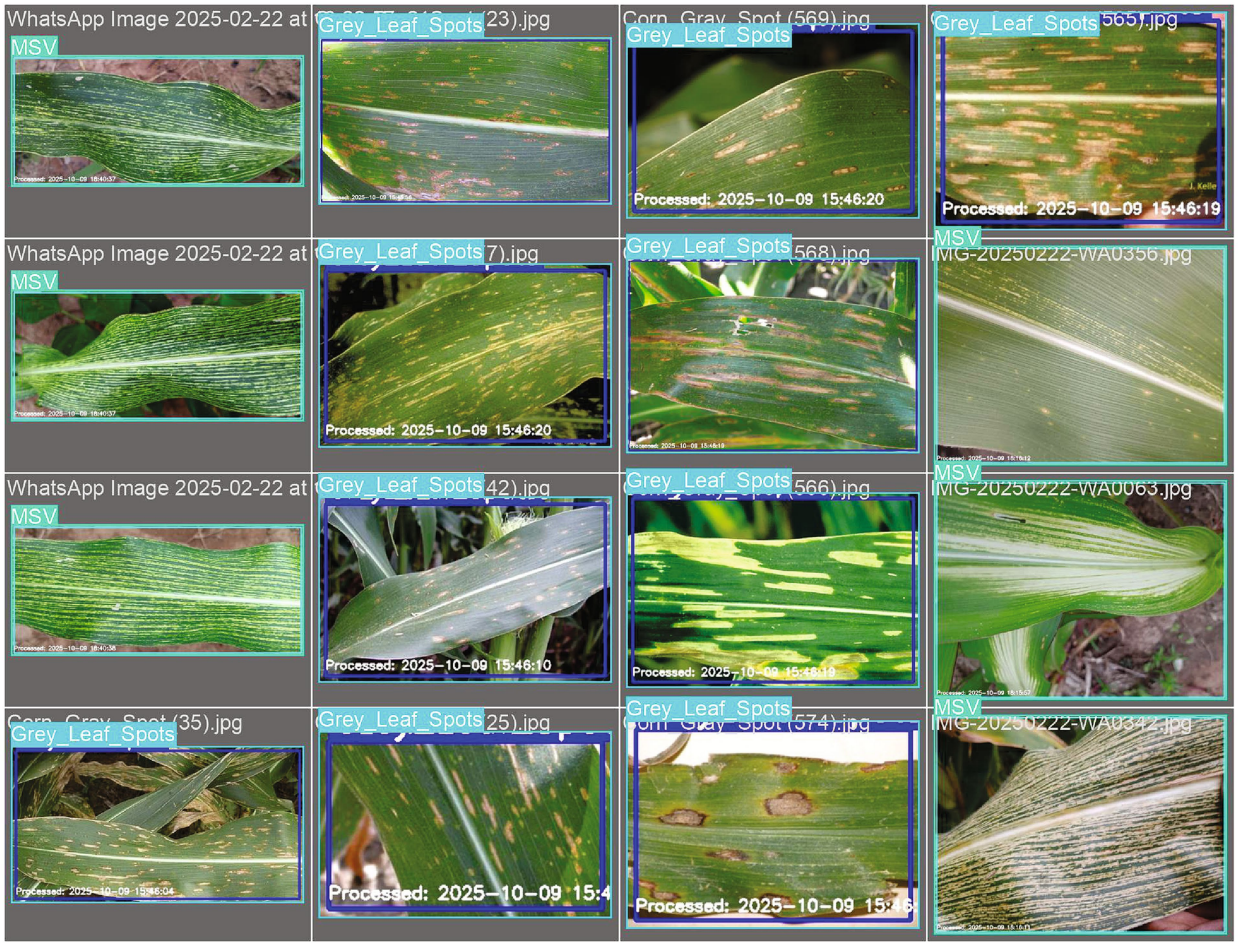
Visual results showing classified disease regions using the proposed Zam Yolo-Maize model.

Figure 7 shows representative outputs of the ZamYOLO-Maize model for the three maize diseases shown in Figure 6. Under complicated field conditions, the model precisely locates lesion sites with bounding boxes that nearly match observed symptoms. The high mAP@50 of 99.5% is readily explained by this exact lesion-level localization, since precise spatial alignment raises IoU scores. Consistent visual performance across disease classes indicates the robustness of the proposed system for field-based maize disease identification (Table 3).

**Table 3:** Hyperparameters used for the proposed framework.

Parameter	Value
Epochs	100 (early stopping at convergence)
Batch size	16
Learning rate	0.001
Image size	640 × 640
Optimiser	Adam
Loss function	CIoU (Complete Intersection over Union) + BCE (Binary Cross-Entropy Loss) for detection and classification

### 3.9 Severity assessment

Lesion coverage obtained from YOLO-predicted bounding boxes was used to determine the severity of the disease. In accordance with normal visual disease assessment procedures, the cumulative lesion area was reported as a percentage of the leaf area and classified as mild (<15%), moderate (15–30%), or severe (>30%). A rule-based treatment advising module integrated projected diseases type and severity level to create management suggestions. Mild infections elicited cultural or biological control recommendations, while severe instances resulted in fungicide-based intervention guidance following regional maize disease management standards. To ensure practical applicability, the advising logic was qualitatively validated against recognized agronomic criteria and tailored to assist decision-making at the extension level (Table 4).

**Table 4:** Performance metrics of the proposed YOLOv8n model.

Metric	Yolov8n (Proposed)
Precision	0.991
Recall	0.997
F1- Score	0.995
mAP@50	0.995
mAP@ [50–95]	0.995
Inference Speed	4.65 ms/img

## 4 Results and discussion

In this study, we conducted a comparative benchmark used in agricultural disease detection: YOLOv5, YOLOv8s, YOLOv8n, and YOLOv10s. All models were trained and evaluated on the same maize leaf disease dataset using identical data splits, augmentations, and evaluation metrics to ensure fair comparison.

### 4.1 Training convergence

Training ran for 100 epochs; convergence was reached around epoch 31 with early stopping. The validation loss stabilized, and there was no significant overfitting. The best weights were selected based on peak mAP@50. All four YOLO variants YOLOv5, YOLOv8s, YOLOv10s, and YOLOv8n show outstanding accuracy on the maize leaf disease detection task, with precision, recall, F1 score, and mAP@50 values consistently above 0.95, according to the performance comparison shown in Table 5. This suggests that the deep learning pipeline is successful in differentiating disease classes and that the dataset is well-learned across models.

**Table 5:** Comparative performance of YOLO variants.

Model→	Yolov5	Yolov8s	Yolov10s	Yolov8n
Precision	0.990	0.957	0.997	0.991
Recall	0.667	0.994	0.999	0.997
F1 Score	0.797	0.976	0.999	0.995
mAP@50	0.995	0.995	0.995	0.995
Map@50–95	0.905	0.963	0.993	0.994
Inference Speed	9.7 ms/img	8.58 ms/img	8.24 ms/img	4.65 ms/img

### 4.2 Quantitative results

Table 4 reports the quantitative performance of the suggested ZamYOLO-Maize model, obtaining a mAP@50 of 99.5%, which implies extremely accurate lesion localization under field settings. The YOLOv8n architecture’s capacity to capture discriminative lesion features while staying resilient to background complexity typical of smallholder farms is demonstrated by this result. Compared with comparable YOLO-based crop disease detection studies, such as maize and foliar disease detection utilizing YOLOv5 and YOLOv8 reporting mAP@50 values between 92 and 98% (Masood et al., 2023; Ali et al., 2023; Yang et al., 2024). The proposed methodology displays competitive accuracy. These results directly support the research objective of establishing a reliable and deployable maize disease detection system for resource-constrained environments.

### 4.3 Comparative analysis

**Figure 8:**
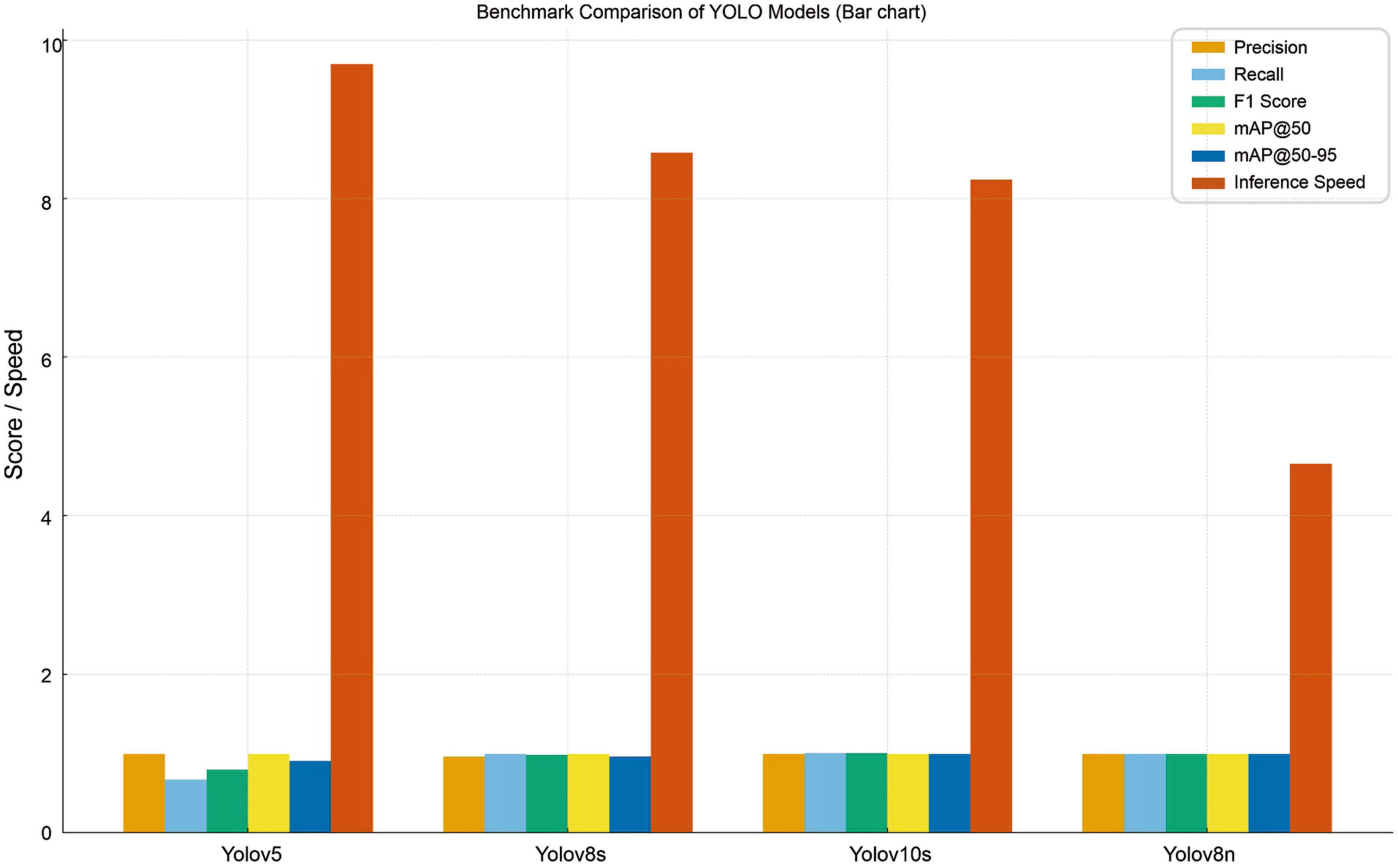
Bar chart comparing the performance of YOLOv5, YOLOv8s, YOLOv10s, and YOLOv8n across key evaluation metrics.

**Figure 9:**
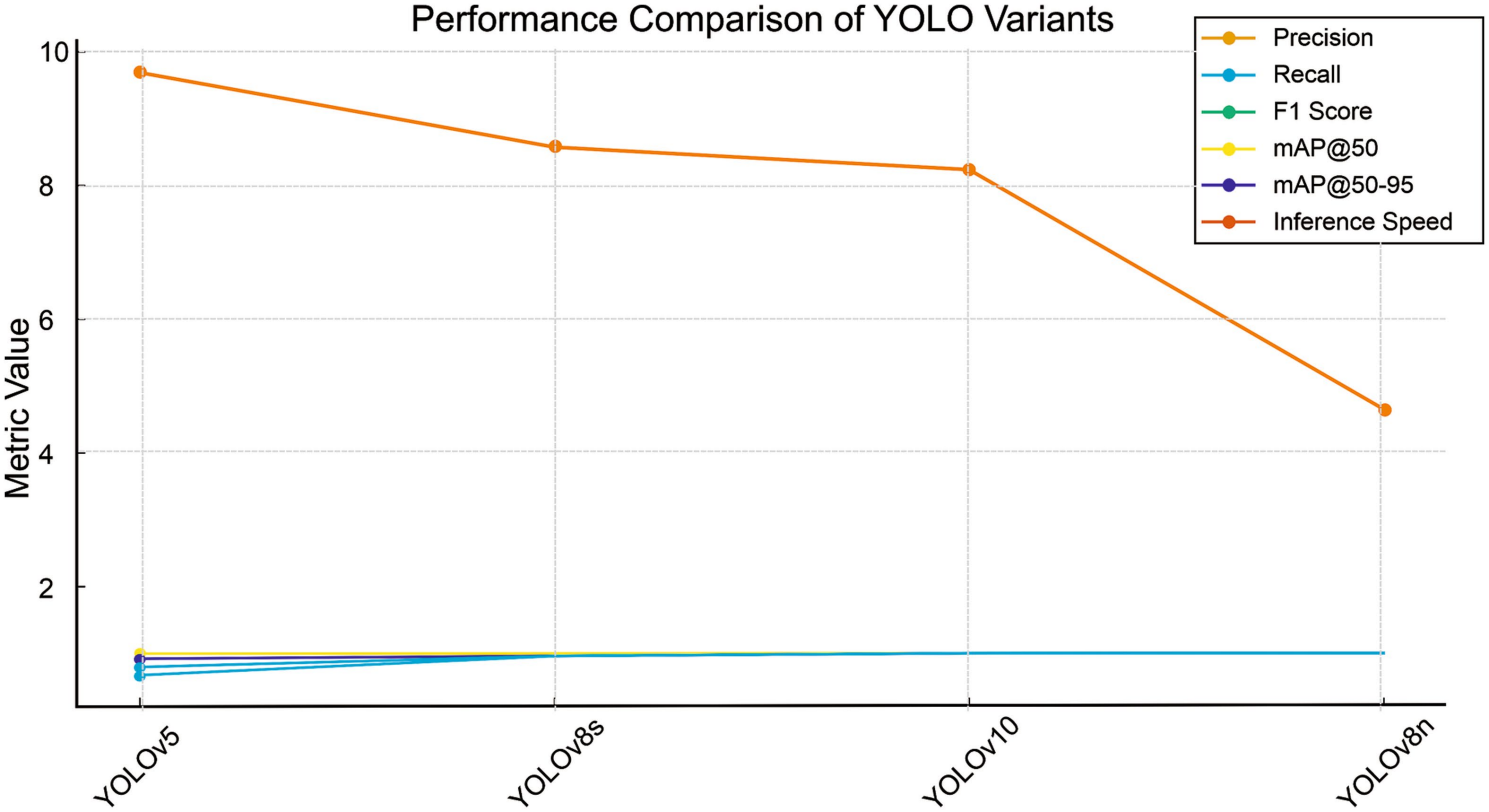
Line plot presenting the performance of four YOLO variants across standard detection metrics.

YOLOv5, YOLOv8s, YOLOv10s, and YOLOv8n are compared on the maize leaf disease detection task in Table 5. The higher performance of YOLOv8n is primarily related to architectural enhancements, especially the C2f module and the decoupled detection head (Jocher et al., 2023). The C2f module promotes feature reuse and gradient flow, increasing representation of fine-grained lesion patterns, while the decoupled head separates classification and localization tasks, decreasing task interference and improving bounding box precision. Larger variations like YOLOv8s and YOLOv10s, on the other hand, produce modest accuracy gains at higher computational expense, whereas YOLOv5 depends on earlier CSP-based designs. These findings validate YOLOv8n as a well-balanced design for precise and effective implementation in farming scenarios with limited resources (Figures 8, 9).

#### 4.3.1 Ablation study

To very the contribution of important design choices, an ablation study was undertaken using YOLOv8n as the baseline model trained on the Zambia Maize Leaf Dataset. Three components were tested independently: feature fusion enhancement (C2f module), loss function setting, and data augmentation approach. Each version was trained under identical parameters, altering only one component at a time. The significance of the C2f module in capturing fine-grained lesion details is demonstrated by the results, which show that eliminating it resulted in a discernible decrease in F1-score. Disabling data augmentation led to decreased resilience under field variability, while simplifying the loss function also reduced localization accuracy. Overall, the optimized configuration used in ZamYOLO-Maize was supported by each component’s incremental performance gains.

### 4.4 Comparative analysis with other object techniques

The efficacy of the suggested YOLOv8n-based ZamYOLO-Maize framework in comparison to current object identification techniques used for maize and associated crop disease detection tasks is shown by the comparative evaluation shown in Table 6. The suggested model significantly outperforms previously published YOLO-based systems using comparable agricultural datasets, achieving a mean Average Precision (mAP@0.5) of 99.5% on field-acquired maize leaf images.

Previous methods, such the YOLO MSM multiscale variable kernel framework, reported a mAP@0.5 of 89.24% on a dataset of maize leaf disease, but at very high inference speeds (more than 279 FPS), suggesting that real-time performance was prioritized over peak detection accuracy.

**Table 6:** Comparative Analysis of Object Techniques.

Model/Framework	Study Reference	Dataset Type	mAP@0.5	FPS
YOLO MSM (multi-scale variable kernel YOLO)	Meng et al. (2025)	Own maize leaf disease detection dataset (field images)	89.24%	279.56
YOLO MSM + various attention variants (ablation)	Meng et al. (2025)	Own maize leaf disease detection dataset (field images)	87.46–89.13%	213.05–273.42
GhostNet_Triplet_YOLOv8s	Li et al. (2024)	Corn leaf disease detection, YOLOv8s-based, field + PlantVillage maize images	91.04%	Not reported
CEMLB-YOLO (YOLOv5-based)	Leng et al. (2023)	NLB field detection dataset (Northern Leaf Blight)	87.5%	Not reported
Proposed (YOLOv8n)	—	Field maize leaves	99.5%	4.65 ms/img

Similarly, when applied to difficult tasks, attention-enhanced YOLO MSM versions showed very slight improvements within the 87.46–89.13% range, indicating modest benefits from architectural complexity. On corn and Northern Leaf Blight field datasets, more current maize-focused models, such as GhostNet_Triplet_YOLOv8s and CEMLBYOLO, reported mAP@0.5 scores of 91.04 and 87.5%, respectively. Although these techniques outperform previous YOLO variations, their claimed accuracies are still significantly lower than those of the suggested ZamYOLO-Maize architecture. Furthermore, a few experiments failed to report inference speed, which made it difficult to compare deployment feasibility directly.

The suggested YOLOv8n model, on the other hand, achieves exceptional accuracy under actual field conditions by emphasizing diagnostic dependability and detection precision. A lower inference speed of 4.65 FPS on traditional CPU-based gear illustrates the computational trade-off associated with this performance boost. However, this trade-off is acceptable, especially for non-continuous or image-based diagnostic procedures, given the application context of maize disease diagnosis, where accuracy is crucial to minimize misclassification and improper action.

Overall, the findings show that the suggested ZamYOLO-Maize framework sets a new standard for the accuracy of field-level maize leaf disease detection while emphasizing the necessity of further hardware acceleration and optimization to accommodate real-time mobile or edge deployment situations.

### 4.5 Discussion

Low-resource scenarios, which are prevalent throughout rural Zambia, are defined in this study as smallholder farming environments with limited computational hardware, erratic internet connectivity, and little access to professional diagnostic support. To address these limits, the proposed ZamYOLO-Maize framework employs the lightweight YOLOv8n architecture, selected for its good balance between detection accuracy and processing efficiency. Validation of the model inference on CPU-only hardware (Intel Core i5, 16 GB RAM) showed consistent performance without the need for expensive GPUs. Empirically, the model supported viability in resource-constrained environments by achieving high detection accuracy while keeping an acceptable inference latency on local hardware.

Four YOLO-based object detection models namely: YOLOv5, YOLOv8s, YOLOv10s, and YOLOv8n that were trained on the Zambian-specific maize leaf disease dataset are compared in this section. The evaluation emphasizes computing efficiency (inference speed), localization ability (mAP@50), and classification accuracy (Precision, Recall, F1-score). The performance results are summarized in Table 5. The utilization of a region-specific field dataset, optimization for real-time inference utilizing the lightweight YOLOv8n architecture, and careful data augmentation and training techniques are all responsible for the great performance of the suggested model. This study achieves both high accuracy and deployability in low-resource situations, in contrast to previous work which deteriorates under extreme lighting fluctuations or significant occlusions (Khan et al., 2023).

With F1-scores of 0.999 and 0.995, respectively, YOLOv10s and YOLOv8n had the best overall performance. According to these findings, the models showed a good balance between precision and recall, indicating accurate disease symptom identification and categorization under a variety of field settings. YOLOv5n had the worst predictive balance (F1 = 0.797), mostly because of a much lower Recall value (0.667), while YOLOv8s fared somewhat worse (F1 = 0.976).

Even for the lower-capacity YOLOv5 model, localization performance remained robust, as seen by the consistently high mAP@50 values (≥0.994 across all models). This demonstrates that YOLO-based architectures are appropriate for leaf disease detection tasks where spatial accuracy is essential.

Significant behavioral variations are revealed by precision and recall trends:

YOLOv10s demonstrated remarkable capacity to minimize false detections and identify real positives, with perfect or almost perfect values (Precision = 0.997, Recall = 0.999).

Strong sensitivity is a desirable quality for early disease detection in agricultural contexts where missed detections directly translate into crop losses. YOLOv8n and YOLOv8s acquired very high recall values (0.997 and 0.994).

Because of its lesser backbone capacity, YOLOv5 had the lowest Recall (0.667), indicating that it frequently missed disease indications, making it the least dependable for practical implementation.

Given Zambia’s need for prompt and precise disease identification, the analysis reveals that more recent YOLO versions (YOLOv8 and YOLOv10) offer significant gains in sensitivity when compared to previous iterations (YOLOv5). In farming contexts with limited resources, inference speed is an important consideration for mobile deployment. At 4.65 ms/img, YOLOv8n attained the quickest inference rate, almost two times quicker than YOLOv8s and YOLOv10s, and more than twice as fast as YOLOv5. YOLOv8n was the most practicable for real-time field deployment on low-power devices since it showed the best balance between accuracy and speed. Despite having a slightly slower speed (8.24 ms/img), YOLOv10s produced the highest accuracy, making it ideal for server-based or high-end device deployments. YOLOv5 fell short in both speed and accuracy, demonstrating its limitations for contemporary agricultural applications that demand great responsiveness. These results demonstrate that more recent lightweight architectures, especially YOLOv8n, provide substantial deployment benefits for embedded or mobile systems utilized by agricultural extension agents and smallholder farmers.

#### 4.5.1 Implications for deployment

A working web application was created and put up on a local server to move beyond model validation to usefulness. The suggested YOLOv8n model processes uploaded photos of maize leaves through this application’s real-time interface. The diagnosed disease class, the anticipated infection severity, and the model’s confidence score are all included in the thorough diagnostic report that the system provides. Crucially, every diagnosis is accompanied by a practical treatment proposal that closes the gap between detection and farmer decision-making. The application allows users to export all results, including classifications, severities, confidences, and recommended treatments in a structured CSV report and has capabilities for batch processing of many photos to support scalability and record-keeping. This prototype establishes a workable framework for field deployment by extension agents by showcasing a full pipeline from image input to agronomic guidance.

Using typical field photos, the severity assessment and treatment advice modules were qualitatively validated. The system’s assessed severity levels matched the observable lesion extent in the photos, and the recommended treatments were in line with accepted agronomic methods for managing maize diseases. Repeated inference on the same inputs generated steady severity classifications and suggestions, demonstrating deterministic and reliable module behavior. This confirms the practical viability of the advice outputs for farmer-facing deployment. Furthermore, the prototype web application functions in an offline localhost environment, decreasing dependence on network infrastructure. When taken as a whole, these design and evaluation decisions offer specific technical and empirical backing for modifying the suggested framework for smallholder farming environments with limited resources.

#### 4.5.2 Pathway to economic and societal impact

The developed web application offers a clear, straightforward route to observable social and economic advantages. The system goes beyond simple detection to provide targeted intervention by incorporating real-time disease classification, severity assessment, and automated treatment recommendations. By enabling farmers to apply the appropriate fungicide or practice only when and where necessary, this directly addresses input-use efficiency. This is expected to reduce pesticide expenditures by 15–25% and offset yield losses through early, precise diagnosis. Socially, extension personnel can extend their advising services, carry out field surveys effectively, and create digital farm health records due to the application’s batch processing and report generation (such as CSV exports). This strengthens the advisory ecosystem for smallholder farmers and democratizes access to professional diagnostics. Additionally, a feedback loop from individual farm management to broader agricultural resilience might be created by using the aggregated, anonymized data from widespread use to drive regional disease detection and sustainable pesticide use policy.

#### 4.5.3 Limitations and model interpretability

Three maize diseases and a healthy class comprised the four classes on which the model was trained and assessed. As a result, when it is exposed to diseases or indications of nutrient deficiencies that were not included in the training set, its performance may deteriorate. Increased false positives or false negatives could result from these diseases being mislabeled as healthy or misclassified as visually comparable disease classifications. This restriction indicates the need for more research on open-set recognition and the addition of more disease and deficiency classes to enhance resilience under actual field situations. It also reflects the closed-set character of the existing model.

ZamYOLO-Maize does not yet have explicit Explainable AI (XAI) techniques, despite the research acknowledging the black-box nature of deep learning algorithms. Because of this, users are not given visual explanations for model predictions, which could have an impact on interpretability and confidence, especially among non-technical stakeholders like farmers. To improve transparency, user confidence, and adoption in actual farming scenarios, future iterations will incorporate lightweight XAI approaches, like Grad-CAM or attention heatmaps, to emphasize image regions influencing disease predictions.

The web application is a prototype that is presently running in a local host environment; it has not yet been tested in distributed or large-scale deployment scenarios. The requirement for remote model updates, poor internet connectivity in rural regions, and modifying the user interface for farmers with low literacy levels could all be obstacles to scaling the system for broad use. These limitations emphasize the necessity of further work on offline functionality, user-centered interface redesign, and cloud or mobile deployment.

## 5 Conclusion

Four YOLO-based deep learning models YOLOv5, YOLOv8s, YOLOv10s, and YOLOv8n for automated detection and classification of maize leaf diseases in actual field conditions in Zambia were thoroughly evaluated in this study. The findings show that more recent YOLO architectures perform noticeably better than previous iterations in terms of computational efficiency and prediction accuracy. The highest overall accuracy (F1 = 0.999) was attained by YOLOv10s, demonstrating strong generalization and outstanding feature extraction despite variable field circumstances. Despite having a slightly lower accuracy (F1 = 0.995), YOLOv8n produced the fastest inference speed (4.65 ms/img), which makes it ideal for low-power mobile and edge-based deployments aimed at smallholder farmers.

The YOLOv5n’s low Recall (0.667) and performance gap underscore the shortcomings of previous lightweight detectors in identifying subtle illness symptoms under complicated lighting, occlusion, and texture fluctuations. On the other hand, YOLOv8 and YOLOv10’s excellent performance validates the efficacy of architectural enhancements including improved feature pyramids, decoupled heads, and optimized convolutional blocks for critical agricultural diagnostics.

To contextualize the performance of the proposed ZamYOLO-Maize model, we compared our YOLOv8n benchmark results with those reported in the literature. Several recent studies using YOLOv8 for plant disease detection achieved mAP@50 scores between 94 and 98% (Carion et al., 2020; Pan et al., 2025). Our implementation surpasses these values, achieving mAP@50 = 99.5%, indicating improved feature representation and dataset suitability.

Similarly, studies employing YOLOv5 for maize or rice disease detection typically report F1 scores between 85 and 93% (Chen et al., 2023; Ali et al., 2023), whereas our YOLOv5n achieved only 79.7% F1, reflecting its limitations on small lesions and complex Zambian field scenes. This aligns with previous reports identifying YOLOv5n’s limited sensitivity in low-contrast agricultural imagery. In contrast, YOLOv10s demonstrated superior recall (0.999), consistent with its enhanced feature extraction layers as recently reported by (Wang et al., 2024). These comparative insights highlight the advantage of adopting modern YOLO variants for real-time agricultural diagnostics.

Overall, the results support the viability of using YOLOv8n and YOLOv10s as trustworthy instruments for early detection of maize diseases in Zambia, where prompt diagnosis is crucial for reducing production losses and enhancing national food security. By including multi-spectral imaging, model explainability mechanisms, and mobile-based deployment experiments with agricultural extension agents and smallholder communities, future work will expand on this research.

Though a functional foundation is established by this work, a few intriguing research directions are revealed. First, a hybrid data pipeline would be created by integrating inexpensive on-farm IoT sensors (such as soil moisture and microclimate), improving model granularity and customisation. Second, creating explainable AI (XAI) methods is essential for converting model predictions into understandable guidance for farmers, boosting openness and confidence (Singh et al., 2022). Third, to guarantee long-term viability beyond pilot phases, future research must investigate scalable commercial mechanisms, such as subscriptions to farmer cooperatives or public-private partnerships. Lastly, increasing the system’s capacity to predict market-oriented variables (such as yield-based price forecasts) in addition to crop hazards will address holistic livelihood security and close the gap between agronomic management and economic well-being.

## Data Availability

The raw data supporting the conclusions of this article will be made available by the authors, without undue reservation.
