# Supplementary material for: ZamYOLO-maize: a YOLOv8n-based deep learning framework for automated detection and classification of maize leaf diseases in field conditions in Zambia
Source: Front Artif Intell. 2026 Feb 25;9:1764283. doi: 10.3389/frai.2026.1764283 (PMC12976002; doi:10.3389/frai.2026.1764283)
Supplement: Supplementary file 1 [file Table_1.DOCX]

Table A1. Ablation analysis of key components in the ZamYOLO-Maize framework on the Zambia Maize Leaf Dataset

| **Configuration** | **Feature Fusion (C2f)** | **Data Augmentation** | **Loss Configuration** | **F1-score** | **ΔF1** |
| --- | --- | --- | --- | --- | --- |
| Baseline YOLOv8n | x | x | default | 0.91 | - |
| + Data Augmentation | x |  | default | 0.93 | +0.02 |
| + Optimized Loss | x |  |  | 0.94 | +0.03 |
| Full ZamYOLO-Maize |  |  |  | 0.96 | +0.04 |
